# Incentives for pregnant mothers during antenatal care for better maternal and neonatal health outcomes: A systematic review protocol

**DOI:** 10.12688/f1000research.109726.1

**Published:** 2022-04-05

**Authors:** Ramesh Holla, Bhaskaran Unnikrishnan, Ratheebhai Vijayamma, Bhumika T V, Anju Sinha, Darshan BB, Rekha T, Prasanna Mithra P, Nithin Kumar, Vaman Kulkarni, Ravishankar N, Rosemol Johnson K

**Affiliations:** 1 Department of Community Medicine, Kasturba Medical College, Mangalore, Manipal Academy of Higher Education, Manipal, Karnataka, India; 2 School of Communication, Manipal Academy of Higher Education, Manipal, Karnataka, India; 3 Public Health Evidence South Asia, Prasanna School of Public Health, Manipal Academy of Higher Education, Manipal, Karnataka, India; 4 Division of Reproductive, Maternal and Child Health, Indian Council of Medical Research, Ansari Nagar, New Delhi, India; 5 Department of Community Medicine and Family Medicine, All India Institute of Medical Sciences, Bibinagar, Telangana, India; 6 Department of Biostatistics, Vallabhbhai Patel Chest Institute, University of Delhi, New Delhi, India

**Keywords:** Pregnancy, Incentives, trials, Maternal outcomes, neonatal outcomes

## Abstract

**Background** **: **Universal access to maternal new-born and child healthcare services (MNCH) is detrimental for attainment of Sustainable Development Goal (SDG) three pertaining to promotion of health at all ages. Incentivization in the form of cash, vouchers, and goods have been used as part of strategies to improve maternal and neonatal health outcomes around the world. However, there exists uncertainties regarding the effectiveness of various incentive-based programmes targeted for pregnant mothers in low- and middle-income countries during their antenatal period.

**Methods: **We will search six electronic databases, namely the Medical Literature Analysis and Retrieval System Online (Medline), Cochrane Central Register of Controlled Trials (CENTRAL), the Cumulative Index to Nursing and Allied Health Literature (CINAHL), Scopus, Web of Science, and Embase in addition to Google Scholar. Manual searching of the reference lists of included studies will also be done. The reporting of this protocol will follow the guidelines of the Preferred Reporting Items for Systematic Reviews and Meta-Analysis Protocols (PRISMA-P) 2015 statement [29]. Only interventional studies that follow randomized, quasi randomized, and cluster randomized controlled study designs will be included. A three-stage screening process will be adopted to select articles. Risk of bias for the included studies will be assessed using the tools and criteria specified in the Cochrane handbook. In addition, the GRADE approach will be used to assess the quality of evidence for the maternal and neonatal health outcomes.

**Conclusion:** This review of trials is essential to inform the effectiveness of incentive-based programmes targeted for pregnant women in low- and middle-income countries. It will help the policy makers to utilise the resources more effectively and to integrate the evidence based public health initiatives into the health system. This can also help build the continuum of care financial packages for all pregnant women.

## Introduction

Around 86% of the maternal deaths in the world occur in South Asia and Sub-Saharan Africa. Most of these deaths occur at home owing to lack of medical attention and are largely preventable. ^1^

The maternal mortality ratio in India as per UNICEF’s data warehouse was 145 per 100,000 live births in 2017 and 113 per 100,000 live births as per the sample registration system (SRS) 2016-2018. ^1^^,^^2^ The neonatal mortality rate in India reduced from 38 deaths in the year 2000 to 22 deaths per 1,000 live births in 2019. ^3^

Given the burden of maternal and neonatal deaths globally, it is essential to implement effective strategies adapted to developing economies that can help achieve the targets of the Sustainable Development Goals (SDG) pertaining to Maternal and child health (MCH). ^4^ In the context of developing economies, many disincentives exist that act as barriers to seeking healthcare for the pregnant woman. These include cultural beliefs, social norms, lack of financial support, attitude of health workers, migration, and language barriers, etc. Customs like ‘Kuthimba’ in Malawi where the woman waits for advice from counsellors from the husband’s side of the family before starting antenatal care (ANC) are rooted in culture. ^5^ An analysis conducted in South Asia and Sub Saharan Africa indicated that women with higher autonomy to make decisions were able to receive the elements of continuum of care compared to those who had no autonomy in decision making. ^6^ Moreover, logistic factors like uneven roads in rural areas and long distance travel to health facilities can further widen the gap in access to health services for pregnant women based on a cross sectional study conducted in Cambodia. ^7^

For an effective continuum of care, it is essential to strengthen the link between home, the primary health care facility and the referral centres. ^8^

Various studies across the globe have shown that health services utilization has considerably increased owing to the Conditional Cash transfer (CCT) initiatives. The Janani Suraksha Yojana was launched in 2005 to promote institutional deliveries in India and thereby reduce neonatal and maternal deaths. As part of this scheme, pregnant women from low socio-economic background receive cash incentives subject to registration at health centres and are encouraged for institutional deliveries. ^9^ The scheme has been instrumental in improving the utilisation of health centres below the district level by pregnant mothers.

Various other intervention studies in the past decade have assessed the impact of types of incentives among pregnant women across the globe. A trial conducted in the Democratic Republic of Congo (DRC) region has found that conditional cash transfers can help achieve retention of pregnant women in care services to prevent mother to child transmission. ^10^ Another pilot, randomised-controlled trial study conducted in Cape Town, South Africa found that participants enrolled in the incentive-based intervention were more likely to attend the clinics delivering antenatal health care services for their first visit before five months of gestation. Also, they were more likely to go to these clinics more than four times. The intervention consisted of a package called the ‘Thula Baba Box’, that contains essential items for clothing and hygiene for babies including items for mothers like maternity pads and condoms. ^11^

These incentive-based interventions are intended to increase uptake of antenatal care services and thereby improve health outcomes. They are also part of behaviour change interventions for smoking/tobacco cessation, improving dietary behaviour among pregnant women.

A previously conducted systematic review has shown only limited evidence that incentives may improve the frequency of prenatal care. This evidence is based on five trials and participants majorly drawn only from low-income communities in Central America and North America. ^12^

In our review, the central component will be incentives. We intend to accumulate evidence pertaining to various incentive-based intervention studies targeted for pregnant women only during the antenatal period in low- and middle-income countries (LMICs) and evaluate the effectiveness across a wide range of outcome measures.

The continuum of care approach for MCH primarily takes into consideration the time of provision of care services. Secondly, it considers the appropriate place of receiving care alongside the various approaches of caregiving services. ^13^ The time dimension can be measured by the number of ANC care visits made by the pregnant mother and also by the components of services received. While the place dimension of the continuum of care looks into the services provided in primary and referral centres and is important to address complications of pregnancy, preterm deliveries, stillbirths and other signs of danger where specific care is required.

This review of trials is thus essential to inform the effectiveness of incentive-based programmes targeted for pregnant women in LMICs. It will help the policy makers to utilise the resources more effectively and to integrate the evidence based public health initiatives into the health system. This can also help build the continuum of care financial packages for all pregnant women.

Our research questions are as follows:

1. Does provision of incentives to pregnant mothers during the antenatal care period achieve better maternal and neonatal health outcomes than the absence of such services for pregnant women?

1a. What are the effects of incentives (any type) on uptake of antenatal care services/utilization of antenatal health care services (frequency of antenatal care, proportion of institutional delivery etc)?

1b. What are the effects of incentives (any type) on maternal and neonatal morbidity (Proportion of preterm deliveries, low birthweight (less than 2,500 g), proportion of antenatal and postnatal complications, compliance to iron-folic acid (IFA) tablets intake and tetanus (TT) injection coverage, and coverage and utilization of maternal incentive based nutritional interventions)?

1c. What are the effects of incentives (any type) on maternal and neonatal mortality (neonatal deaths/maternal deaths)?

### Objective of the systematic review and meta-analysis

• To determine if any of the incentive-based interventions had an effect on maternal outcomes (Proportion of antenatal and postnatal complications, proportion of institutional delivery, frequency of antenatal care, maternal deaths, compliance to IFA tablets intake and TT injection coverage, coverage and utilization of maternal incentive based nutritional interventions, and cessation of smoking, alcohol, tobacco, or any other unhealthy behavioural practices).
• To determine if any of the incentive-based interventions had an effect on neonatal health outcomes (Proportion of preterm deliveries, low birthweight (less than 2,500 g), and perinatal and neonatal deaths).

### Eligibility criteria

*Participants*

We will include all incentive based interventional studies conducted on pregnant women in lower-middle income countries (LMICs) as per the World Bank definition. ^14^ We will exclude studies conducted among pregnant women that belong to any of the low- and middle-income countries but reside in high income countries as immigrants during the antenatal and postnatal period. We will exclude studies that involve pregnant women living in high income countries but belonging to low-income families/communities. We will exclude studies that are focused on incentives for healthcare providers, government agencies or any other stakeholders on the supply side.

*Intervention*

We will conduct a systematic review of all interventions that include incentives given to pregnant mothers linked to their antenatal care, which are usually not offered to pregnant mothers as a standard prenatal care. We will not place any restriction with regard to the modes of financing. These could be conditional cash transfers, vouchers, transport services, in-kind goods, mama kits, co-payments, etc. We will also include incentive-based behaviour change intervention studies conducted during the antenatal care period among pregnant women for incentive-based smoking/tobacco cessation interventions, incentivised nutrition focussed interventions for appropriate dietary behaviour during pregnancy, etc.

*Comparators*

The interventions of our interest can be compared to routine antenatal care (no incentives), no intervention or any other type of intervention that is not considered as incentives. We will not restrict the definition of usual care/routine antenatal care.

*Outcomes*

The outcomes of interest are categorized as ‘neonatal outcomes’ and ‘maternal outcomes’. Neonatal outcomes of interest are as follows:

• Proportion of preterm deliveries.
• Low birthweight (less than 2,500 g).
• Perinatal and neonatal deaths.

Maternal outcomes of interest are as follows:

• Frequency of antenatal care (number of visits and the content of care).
• Proportion of antenatal and postnatal complications.
• Proportion of institutional delivery.
• Maternal deaths.
• Compliance to IFA tablets intake and TT injection coverage.
• Coverage and utilization of maternal incentive based nutritional interventions.
• Cessation of smoking, alcohol, tobacco, or any other unhealthy behavioural practices.

All the above outcomes will be assessed in our review with regard to incentives targeted for pregnant women during the antenatal period only. We will exclude studies that do not measure at least one of the above outcomes.

*Types of study designs*

We will include studies with experimental designs such as randomised, quasi – randomised, and cluster randomised studies. Pilot randomised controlled trials (RCTs) also will be included in this review. Observational studies will be excluded in this review since the review is to determine the effectiveness of incentive-based intervention studies alone. We will restrict our studies to English language publications owing to a lack of resources for translation and nonavailability of multilingual experts. Only studies published in scientific journals will be considered for inclusion. We will exclude editorials, opinion papers, reviews, and case studies. Studies that are published as abstracts and conference proceedings will be included if we can retrieve the full texts from the authors of the trials. In addition, preprints of unpublished studies will be included.

## Methods

We have followed the 17 items listed in the PRISMA-P 2015 checklist for reporting this protocol. ^15^ We will follow the updated PRISMA 2020 guidelines consisting of 27 listed items for reporting our systematic review. ^16^ The conduct of the systematic review will be in adherence to the Cochrane methods as mentioned in the Cochrane Handbook for Systematic reviews on Interventions. ^17^

### Search strategy

We will conduct the search on the following electronic databases using a set of keywords and medical subject headings. We will take the assistance of an information specialist who works as a librarian in the University to develop and run the search strategy. We will utilise her expertise in the same manner. We have listed the search concepts and keywords in table 1 for Medline only. ^28^ We will undertake the search in Medical Literature Analysis and Retrieval System Online (Medline), Cochrane Central Register of Controlled Trials (CENTRAL), Cumulative Index to Nursing and Allied Health Literature (CINAHL), Scopus, Web of Science, and Embase. In addition, we will undertake manual searching on the Google Scholar database using the search [“pregnancy” AND “incentive”] and will download the results shown in the first 20 pages, with each page showing 10 results. ^18^ Reference lists of studies that we include after full text screening will be searched so that we capture more relevant articles. We will not restrict the search to the year of publication. In accordance with the guidelines in the PRISMA statement, we will use the Population, Intervention, Comparator, Outcome (PICO) acronym to inform the search strategy as mentioned in the Cochrane handbook. For study design, we will use the search strategy as specified in Cochrane methods. This strategy has been found to be highly sensitive for identifying randomised controlled trials on Medline. ^19^ For other databases, we will customise the terms accordingly. For Embase, we will use the standardised keywords that are available under ‘Emtree’ terms. Likewise for Medline we will use, the MESH terms. We will also use appropriate field codes and Boolean operators.

We will initiate contacts with authors of published trials for clarification concerning the included studies. We will also do targeted searches with regard to the included trials to identify published protocols, pilot studies, updates, comments and corrections on the trials. We will then export the results of the search to Covidence software ^20^ that facilitates automatic removal of duplicates. The draft search strategy for Medline has been provided in supplemental file 1. ^17^

### Study selection, data collection, and analyses

*Screening articles*

The screening process also will be undertaken on Covidence software. First, two reviewers will independently do the screening of titles followed by abstract screening based on the eligibility criteria specified. Full text screening will be independently performed by two reviewers. After downloading all the full texts for the purpose of full text screening, records that are ineligible will be removed with specific reasons that will be documented in a flow chart. If there are disagreements, a third reviewer will resolve by discussion for the final decision regarding the inclusion of the particular study. The reporting will be as per the guidelines of the Preferred Reporting Items for Systematic Reviews and Meta-Analysis (PRISMA) 2020 statement. ^16^ We will also provide a summary of the excluded trials along with the specific reasons for exclusion. In addition, we will also keep a record of ongoing trial protocols.

*Data extraction*

Two reviewers will independently extract data under the following headings: study ID, author details, publication year, participant details, intervention details, comparators, outcomes, funding details, and any other important details if required. We will independently pilot the data extraction form so as to ensure that all relevant details are captured and modify the data extraction form accordingly. We will initiate contact with authors of included articles for missing information in those studies. Discrepancies in the extracted data between the two reviewers will be resolved by discussion. A third author will be involved to achieve Consensus. The data will be entered into Review Manager, version 5.4 ^21^ by one author and verified by another author.

In order to reduce risk associated with publication bias, we will conduct searches for trials within the previous reviews on Incentives for pregnant women.

*Risk of bias assessment*

Risk of bias will be assessed using the tools mentioned in the Cochrane Handbook for Systematic Reviews of Interventions. ^22^ This assessment will be independently conducted by two investigators. In case of disagreements, a third investigator will be involved for resolution of the same. The current version of the ROB-2 tool (version 2019) will be used for the studies with randomised study designs. The variant of ROB 2 tool adapted for cluster randomised trials (2021 version) will be used for cluster randomised trials. We will evaluate and report the results in the ‘risk of bias’ tables. The following criteria will be used to assess the risk of bias in each of the randomized controlled studies that will be included after full text screening.

• Selection bias (random sequence generation and allocation concealment)
• Performance bias
• Detection bias
• Attrition bias
• Reporting bias

We will categorize the risk of bias for each domain as ‘low’, ‘unclear’, and ‘high’ based on the adequateness of each criterion mentioned in the risk of bias tools specified in the Cochrane handbook. ^22^

The ROBINS-I tool will be used for assessing the risk of bias for quasi randomised controlled trial studies. The tool will provide guidance to cover seven domains of bias (pre intervention, at intervention, and post intervention) and the five judgement responses (low, moderate, serious, critical risk of bias, and no information). ^23^^,^^24^

*Data synthesis*

We will perform the statistical analysis for data analysis on Review Manager Software, version 5.4. ^21^ as per the statistical guidelines referenced in the Cochrane Handbook. ^25^ We will conduct a random effect meta-analysis in the case of moderate to severe heterogeneity or else we will generate fixed effect models. If a high level of heterogeneity is evident, only a narrative summary of trial findings will be provided. We will present the data in a forest plot and summary of findings tables. Results for dichotomous outcomes will be expressed as risk ratios and risk differences with 95% confidence intervals. Calculation of the number needed to treat for additional outcomes will be done in the event that the RD is statistically significant. Measures of effect will be expressed as mean differences with 95% CIs for outcomes that are reported on continuous scales. After standardisation of outcomes, standardised MD will be calculated if the data is reported on different continuous scales. While analysing the RCTs and quasi RCTs, we will check if the randomisation was done at individual or group/cluster level. If cluster-randomised studies are included, accordingly those will be adjusted for clustering. We will multiply the standard error derived from the confidence interval of the effect estimate by the square root of the design effect. We will utilize the generic inverse variance method in Review Manager 5 to perform meta-analysis using inflated variances.

We will use the T ^2^, I ^2^, and Chi ^2^ statistics to assess the heterogeneity in the meta-analysis. In the Chi ^2^ test, if the I ^2^ shows more than 30% or the T ^2^ was more than zero, or the P value is low (<0.10) then the heterogeneity will be considered substantial.

The need for the sensitivity analysis will be decided based on the many decision nodes that we anticipate encountering during the systematic review process. Considering the inclusion of quasi randomized studies, if required, then the sensitivity analysis will be conducted to ascertain the robustness of the results to the ROB in the included studies. The sensitivity analysis will be reported in the form of summary tables rather than individual forest plots.

Subgroup analyses for the maternal and neonatal health outcomes will be conducted. The subgroups will be defined by the intervention type. The effect of the intervention will be estimated for each subgroup.

We will report the effect of the interventions on the specific outcomes mentioned in this protocol. In addition, we will also report about the certainty of these findings as high, moderate, low and very low in accordance with the GRADE levels. ^26^ We will use the GRADE profiler software and guideline development tool to develop the summaries. ^27^ One review author will conduct the assessments for GRADE and another author will verify the assessments. We will present the data on a table where findings will be summarized for the effects of interventions on the specific listed outcomes.

## Discussion

The current review will systematically follow the Cochrane methodology. ^14^^–^^26^ The results generated out of this systematic review will inform or guide in implementing public health interventions and policy with regard to incentive-based initiatives for pregnant women.

No restrictions with regard to the publication year will be followed for the search strategy.

We are including all the modes of financing that were implemented for pregnant women. There will be no restrictions on the above. The modes of financing can include vouchers, transport incentives, in-kind goods, cash transfers, etc.

Inclusion of studies will be restricted to English language and those published in scientific journals.

## Data availability

### Underlying data

Figshare: Medline database search strategy for ‘Incentives for pregnant mother during antenatal care for better maternal and neonatal health outcomes: A Systematic Review Protocol’. https://doi.org/10.6084/m9.figshare.19161494.v3 ^28^

This project contains the following underlying data:

• Supplemental File 1: Search Strategy for Medline database

Data are available under the terms of the Creative Commons Zero “No rights reserved” data waiver (CC0 1.0 Public domain dedication).

### Reporting guidelines

Figshare: PRISMA-P checklist for ‘Incentives for pregnant mother during antenatal care for better maternal and neonatal health outcomes: A Systematic Review Protocol’.

https://doi.org/10.6084/m9.figshare.19161413.v3. ^29^

Data are available under the terms of the Creative Commons Zero “No rights reserved” data waiver (CC0 1.0 Public domain dedication).

## Authors contribution

RH conceived and designed the study. RH secured funding for this systematic review and is the guarantor of the systematic review. RH, RJ, BTV drafted the manuscript. RV, RH, RJ developed the search strategy, research questions and study design. NK, RT, PM, DB, VK, RJ, RH, designed the tables for data extraction, will perform data extraction, and also evaluate the quality of all included studies in the systematic review. RH, RV, RJ, BTV contributed to the introduction section and supplementary files 1 and 2. RT, PM, RN, DB will contribute to data synthesis and meta-analysis. AS, BTV and BU provided direction, mentorship and extensively revised the manuscript. All the authors are in consensus to abide by the protocol and ensure their services throughout the conduct of the review process.

**Study status:** This study is currently at the stage of data synthesis.
