# Supplementary material for: Incentives for pregnant mothers during antenatal care for better maternal and neonatal health outcomes in low and middle income countries: A systematic review protocol
Source: F1000Res. 2022 May 13;11:393. Originally published 2022 Apr 5. [Version 2] doi: 10.12688/f1000research.109726.2 (PMC9160705; doi:10.12688/f1000research.109726.2)
Supplement: Peer review history

# Peer review: Reviewer response for version 2

**DOI:** 10.5256/f1000research.133955.r137715

**Published:** 2022-06-01

**Authors:** Preethy D'Souza

**Affiliations:** 1 Social Sciences Research Unit, Department of Social Sciences, University College London Institute of Education, London, UK

I have gone through the response from authors for my comments about the protocol. I am happy about the revisions and I have no further comments to make.

Is the study design appropriate for the research question?

Yes

Is the rationale for, and objectives of, the study clearly described?

Partly

Are sufficient details of the methods provided to allow replication by others?

Partly

Are the datasets clearly presented in a useable and accessible format?

Not applicable

Reviewer Expertise:

Evidence synthesis, Public Health, International Development

I confirm that I have read this submission and believe that I have an appropriate level of expertise to confirm that it is of an acceptable scientific standard.

# Peer review: Reviewer response for version 1

**DOI:** 10.5256/f1000research.121269.r130035

**Published:** 2022-04-29

**Authors:** Preethy D'Souza

**Affiliations:** 1 Social Sciences Research Unit, Department of Social Sciences, University College London Institute of Education, London, UK

Overall well-written protocol on a relevant topic. The authors followed Systematic Review (SR) guidelines.

 However, the review scope is more like a systematic map rather than a synthesis. When I read the protocol, I understand that the review authors are trying to look at different types of incentives on a wide range of outcomes. This could make synthesis less useful and superficial. 

 Hence, my suggestion is to limit the outcomes rather than looking at it very broadly - it will be helpful if the authors could provide more conceptual clarity on interventions and outcomes and how they are going to synthesize the data. It is mentioned that the subgroups will be defined by the intervention type. How about the wide range of outcomes such as mortality, care utilization, service provisions, morbidity, behavioural change etc. will they be grouped for analysis?

Is the study design appropriate for the research question?

Yes

Is the rationale for, and objectives of, the study clearly described?

Partly

Are sufficient details of the methods provided to allow replication by others?

Partly

Are the datasets clearly presented in a useable and accessible format?

Not applicable

Reviewer Expertise:

Evidence synthesis, Public Health, International Development

I confirm that I have read this submission and believe that I have an appropriate level of expertise to confirm that it is of an acceptable scientific standard, however I have significant reservations, as outlined above.

# Response

**Published:** 2022-05-11

**Authors:** Ramesh Holla

**Affiliations:** Kasturba Medical College, Mangalore, Manipal Academy of Higher education, Manipal, India, India

Thank you for your suggestions. We appreciate your time and willingness to review the manuscript.

 As per your suggestions, we have provided conceptual clarity on the outcomes and interventions.

 We will do subgroup analysis based on the outcomes or intervention types depending on the availability of sufficient data from existing literature sources.

  

- In outcomes section, we have added definitions beside the outcome names. For preterm deliveries, we added ‘(baby born alive before 37 weeks)’, perinatal death (death between 28 weeks of gestation to first 7 days of life) and neonatal deaths (death within first 28 days of life), maternal deaths (death during pregnancy and childbirth or within 42 days of termination of pregnancy). 
- We have added a new reference no. 15 for institutional deliveries.
- In addition, for TT vaccination uptake outcome, we have added ‘(operational definition for vaccine uptake in our review is based on if the mother received any number of doses of the TT vaccine)’. 
- We changed the word ‘TT injection coverage’ to ‘TT vaccination uptake’.
- Likewise, we removed the word ‘coverage’ in outcome name ‘coverage and utilization of maternal incentive based nutritional interventions’. So, the new outcome name is 'utilization of maternal incentive based nutritional interventions’.
- In Intervention section, we added this sentence, ‘the incentive-based interventions can be both public health care system driven or initiated by NGOs or others’.
- In Data synthesis section, we have added the statement, ‘we will do subgroup analysis based on the outcomes or intervention types depending on the availability of sufficient data from existing literature sources’.

# Peer review: Reviewer response for version 1

**DOI:** 10.5256/f1000research.121269.r130034

**Published:** 2022-04-29

**Authors:** Praveen Kulkarni

**Affiliations:** 1 Department of Community Medicine, JSS Medical College, JSS Academy of Higher Education and Research, Mysuru, Karnataka, India

The proposed systematic review protocol on incentives for pregnant mothers during antenatal care for better maternal and neonatal health outcomes is a timely and most relevant effort by authors to throw light on a very attractive intervention to improve pregnancy outcomes. The review proposes to focus on the effectiveness of commonly adapted interventions to attract pregnant women to utilize maternal new-born and child healthcare (MNCH) services in low- and middle-income countries (LMICs).

 Few suggestions that can be taken into consideration to further improve the review are as follows:

1. As the review is going to focus on low- and middle-income countries, it can be mentioned in title to make it more explicit.
2. In the inclusion criteria, authors can mention the incentive-based interventions can be both public health care system driven or initiated by NGOs or others.
3. In outcomes can authors define pre-term in the same way they have defined low birth weight (LBW)?
4. Please think of quasi experimental to be added/replaced along with quasi randomized, as most of the studies could be community-based trials, where quasi experimental may sound better than quasi randomized.

Is the study design appropriate for the research question?

Yes

Is the rationale for, and objectives of, the study clearly described?

Yes

Are sufficient details of the methods provided to allow replication by others?

Yes

Are the datasets clearly presented in a useable and accessible format?

Yes

Reviewer Expertise:

Epidemiology, Medical Education, Clinical research

I confirm that I have read this submission and believe that I have an appropriate level of expertise to confirm that it is of an acceptable scientific standard.

# Response

**Published:** 2022-05-11

**Authors:** Ramesh Holla

**Affiliations:** Kasturba Medical College, Mangalore, Manipal Academy of Higher education, Manipal, India, India

Thank you for your suggestions. We appreciate your time and willingness to review the manuscript.

 We have incorporated the following changes in the manuscript based on your suggestions.

1. *"As the review is going to focus on low- and middle-income countries, it can be mentioned in title to make it more explicit."*
**Response**: Added the words ‘low- and middle-income countries’ in the study title.
2. *"In the inclusion criteria, authors can mention the incentive-based interventions can be both public health care system driven or initiated by NGOs or others."*
**Response:** In Intervention section, we added this sentence, ‘the incentive-based interventions can be both public health care system driven or initiated by NGOs or others’.
3. *"In outcomes can authors define pre-term in the same way they have defined low birth weight (LBW)?"*
**Response**: For preterm deliveries, we added ‘(baby born alive before 37 weeks)’.
4. *"Please think of quasi experimental to be added/replaced along with quasi randomized, as most of the studies could be community-based trials, where quasi experimental may sound better than quasi randomized."*
**Response**: Thank you for your critical feedback. Owing to time constraints and scope of the current review, we have decided to limit the review to randomized trials and quasi randomized trials only.
